# The *GBA* p.G85E mutation in Korean patients with non-neuronopathic Gaucher disease: founder and neuroprotective effects

**DOI:** 10.1186/s13023-020-01597-0

**Published:** 2020-11-11

**Authors:** Yoo-Mi Kim, Jin-Ho Choi, Gu-Hwan Kim, Young Bae Sohn, Jung Min Ko, Beom Hee Lee, Chong Kun Cheon, Han Hyuk Lim, Sun-Hee Heo, Han-Wook Yoo

**Affiliations:** 1 Department of Pediatrics, College of Medicine, Chungnam National University, Chungnam National University Sejong Hospital, Sejong, Korea; 2 Department of Pediatrics, College of Medicine, Chungnam National University, Chungnam National University Hospital, Daejeon, Korea; 3 Department of Pediatrics, Asan Medical Center Children’s Hospital, University of Ulsan College of Medicine, 88 Olympic-ro 43-gil, Songpa-Gu, Seoul, 05505 Korea; 4 Medical Genetics Center, Asan Medical Center, University of Ulsan College of Medicine, Seoul, Korea; 5 Department of Medical Genetics, Ajou University School of Medicine, Ajou University Hospital, Suwon, Korea; 6 Department of Pediatrics, College of Medicine, Seoul National University Children’s Hospital, Seoul, Korea; 7 Department of Pediatrics, College of Medicine, Pusan National University Children’s Hospital, Yangsan, Korea

**Keywords:** Gaucher disease, Founder effect, *GBA*, β-Glucocerebrosidase

## Abstract

**Background:**

Gaucher disease (GD) is caused by a deficiency of β-glucocerebrosidase, encoded by *GBA*. Haplotype analyses previously demonstrated founder effects for particular *GBA* mutations in Ashkenazi Jewish and French-Canadian populations. This study aimed to investigate the clinical characteristics and mutation spectrum of *GBA* in Korean GD patients and to identify founder effect of *GBA* p.G85E in non-neuronopathic GD patients.

**Results:**

The study cohort included 62 GD patients from 58 unrelated families. Among them, 18 patients from 17 families harbored the p.G85E mutation. Haplotype analysis was performed for 9 probands and their parents for whom DNA samples were available. In 58 unrelated probands, the *GBA* mutation p.L483P was the most common (30/116 alleles, 26%), followed by p.G85E (16%), p.F252I (13%), and p.R296Q (9%). The median age at diagnosis of the 18 patients harboring the p.G85E mutation was 3.8 (range 1.2–57) years. No patients developed neurological symptoms during follow-up periods of 2.2–20.3 (median 13.9) years. The size of the shared haplotype containing *GBA* p.G85E was 732 kbp, leading to an estimated age of 3075 years.

**Conclusion:**

The *GBA* p.G85E mutation, which appears to be neuroprotective despite producing distinctive visceromegaly and skeletal symptoms, exhibited a potential founder effect in Korean GD patients.

## Background

Gaucher disease (GD [MIM: 230,800, 230,900, and 231,000]) is caused by the deficiency of β-glucocerebrosidase (GBA), encoded by *GBA* [1]. Progressive glycolipid accumulation in the reticuloendothelial system leads to anemia, thrombocytopenia, hepatomegaly, splenomegaly, and skeletal manifestations, such as bone pain, avascular necrosis, and osteoporosis [1, 2]. GD is categorized into non-neuronopathic (type 1), acute neuronopathic (type 2), and chronic neuronopathic (type 3) types, in accordance with the degree of central nervous system involvement [1]. Adult-onset Parkinsonism may occur in patients with type 1 GD [1, 2]. However, Parkinsonism is not considered as a feature of neuronopathic GD; rather, *GBA* mutations are one of the risk factors of Parkinsonism [3].

Mutation spectra differ among ethnicities [4, 5], and genotype–phenotype correlations have been noted in some mutations. According to the International Collaborative Gaucher Group’s Gaucher Registry (clinicaltrials.gov NCT00358943), ~ 94% of patients in Western countries are of the non-neuronopathic type, whereas half of the patients in Japan, Korea, and China are classified as the neuronopathic type [4]. Common mutations including c.1226A > G (p.N409S), c.1448 T > C (p.L483P), c.115 + 1G > A (IVS2 + 1), and c.84dupG (p.L29Afs*18), account for 90% of all mutations in the Jewish population and 50% of all mutations in the non-Jewish populations [3, 6, 7]. Among these, the most prevalent mutation among Caucasians is p.N409S, which accounts for approximately 80% of mutations [8]. GD patients harboring a p.N409S mutation even in one allele can be excluded from a diagnosis of neuronopathic GD (type 2 or 3) [3], suggesting that the mutation may exert a neuroprotective effect [3, 5–7]. However, the p.N409S mutation has been rarely identified among Asian GD patients, particularly among Japanese and Korean patients [4, 8, 9]. Instead, p.L483P and p.F252I mutations are more prevalent among Asian groups, where the homozygosity of these mutations is generally associated with neuronopathic GD [4, 9–11].

Interestingly, a few recurrent *GBA* mutations, including p.N409S in Ashkenazi Jewish and European communities [12], p.D448H in northern Swedish [13], p.W417G in French-Canadian [14], and p.G416S in Tabuleiro do Norte, Northeastern Brazilian [15] populations have been suggested as founder mutations.

The *GBA* p.G85E mutation has been identified in Asian populations, exclusively in Korea, China, and India [4, 9–11, 16]. Furthermore, the p.G85E mutation was found exclusively in non-neuronopathic GD patients [4, 9], suggesting a potential neuroprotective allele. This study aimed to investigate the clinical characteristics of GD patients carrying p.G85E mutation and mutation spectrum of *GBA* among Korean GD patients and to test for a founder effect for the *GBA* p.G85E mutation via haplotype analysis.

## Results

### Mutation spectrum of Korean GD patients

**Fig. 1:**
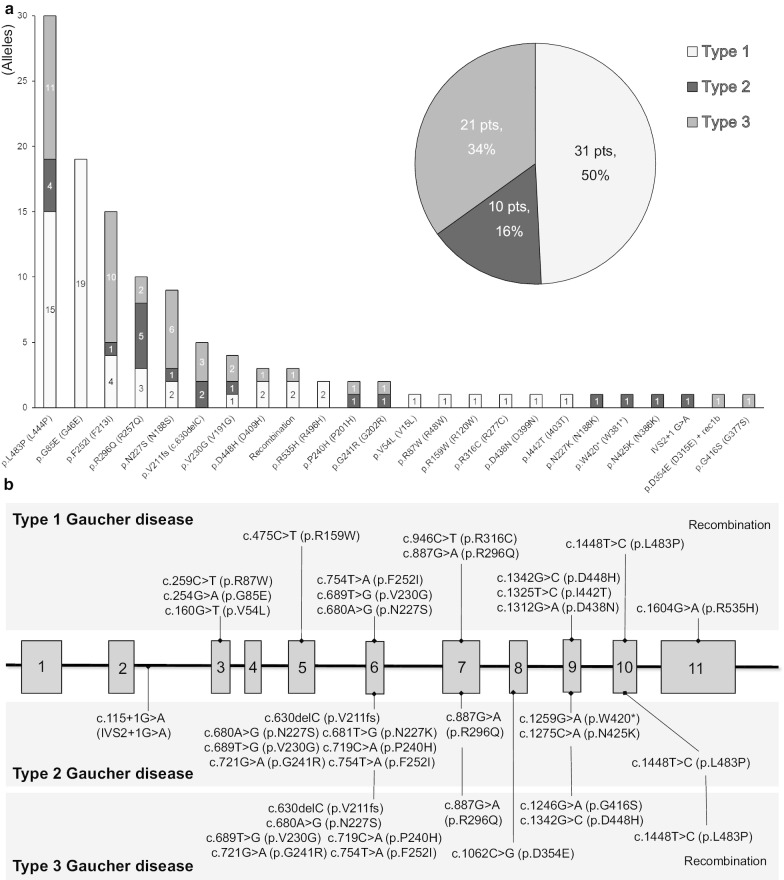
Proportion of type 1, 2 and 3 Gaucher disease and genotype in 62 Gaucher Disease patients from 58 unrelated Korean families (**a**) and distribution of 24 *GBA* mutations according to phenotype (**b**)

Twenty-four different mutations, including 20 missense, one nonsense, one frameshift, one splicing, and recombinants, were identified in 62 patients from 58 unrelated families (Fig. 1a; Table 1). Among the 20 missense variants, 14 pathogenic mutations, and six likely-pathogenic variants (p.V230G, p.P240H, p.R316C, p.D354E, p.N425K, and p.I442T), classified according to American College of Medical Genetics guidelines, were identified.

**Table 1:** *GBA* genotypes of 62 Korean Gaucher disease patients from 58 unrelated families

Subject	Sex	Type	Allele 1	Allele 2	Subject	Sex	Type	Allele 1	Allele 2
1	F	I	p.G85E (exon 3)	p.R159W (exon 5)	32	M	II	p.N425K (exon 9)	p.L483P (exon 10)
2	F	I	p.G85E (exon 3)	p.R296Q (exon 7)	33	F	II	p.F252I (exon 6)	p.P240H (exon 6)
3	F	I	p.G85E (exon 3)	p.R296Q (exon 7)	34	F	II	p.W420* (exon 9)	p.L483P (exon 10)
4	F	I	p.G85E (exon 3)	Rec8a	35	M	II	p.N227S (exon 6)	p.N227K (exon 6)
5	M	I	p.G85E (exon 3)	p.D438N (exon 9)	36	M	II	p.V211fs (exon 6)	p.R296Q (exon 7)
6	M	I	p.G85E (exon 3)	p.F252I (exon 6)	37	M	II	p.L483P (exon 10)	p.V211fs (exon 6)
7^a^	F	I	p.G85E (exon 3)	p.F252I (exon 6)	38	M	II	p.R296Q (exon 7)	p.R296Q (exon 7)
8^a^	M	I	p.G85E (exon 3)	p.F252I (exon 6)	39	F	II	p.L483P (exon 10)	IVS2 + 1 G > A
9	M	I	p.G85E (exon 3)	p.F252I (exon 6)	40	M	II	p.G241R (exon 6)	p.R296Q (exon 7)
10	F	I	p.G85E (exon 3)	p.L483P (exon 10)	41	M	II	p.V230G (exon 6)	p.R296Q (exon 7)
11	F	I	p.G85E (exon 3)	p.L483P (exon 10)	42	F	III	p.V211fs (exon 6)	p.N227S (exon 6)
12	M	I	p.G85E (exon 3)	p.L483P (exon 10)	43	F	III	p.N227S (exon 6)	p.V211fs (exon 6)
13	F	I	p.G85E (exon 3)	p.L483P (exon 10)	44	F	III	p.V230G (exon 6)	p.L483P (exon 10)
14	M	I	p.G85E (exon 3)	p.L483P (exon 10)	45	M	III	p.L483P (exon 10)	p.L483P (exon 10)
15	F	I	p.G85E (exon 3)	p.I442T (exon9)	46	F	III	p.F252I (exon 6)	p.L483P (exon 10)
16	M	I	p.G85E (exon 3)	p.G85E (exon 3)	47	M	III	p.V211fs (exon 6)	p.N227S (exon 6)
17	F	I	p.G85E (exon 3)	p.G85E (exon 3)	48	F	III	p.L483P (exon 10)	p.V230G (exon 6)
18	M	I	p.G85E (exon 3)	p.V230G (exon 6)	49	F	III	p.F252I (exon 6)	p.R296Q (exon 7)
19	M	I	p.V54L (exon3)	p.L483P (exon 10)	50	M	III	p.F252I (exon 6)	p.F252I (exon 6)
20^b^	M	I	p.R87W (exon 3)	p.R296Q (exon 7)	51	M	III	p.F252I (exon 6)	p.L483P (exon 10)
21^b^	M	I	p.R87W (exon 3)	p.R296Q (exon 7)	52	F	III	p.F252I (exon 6)	p.G416S (exon 9)
22	F	I	p.N227S (exon 6)	p.L483P (exon 10)	53^d^	F	III	p.N227S (exon 6)	p.R296Q (exon 7)
23	M	I	p.N227S (exon 6)	p.L483P (exon 10)	54^d^	F	III	p.N227S (exon 6)	p.R296Q (exon 7)
24	F	I	p.F252I (exon 6)	p.L483P (exon 10)	55	M	III	p.F252I (exon 6)	p.F252I (exon 6)
25	F	I	p.R316C (exon 9)	p.L483P (exon 10)	56	F	III	p.P240H (exon 6)	p.L483P (exon 10)
26 ^c^	M	I	p.D448H (exon 9)	p.L483P (exon 10)	57	M	III	p.L483P (exon 10)	p.L483P (exon 10)
27 ^c^	M	I	p.D448H (exon 9)	p.L483P (exon 10)	58	F	III	p.G241R (exon 6)	p.N227S (exon 6)
28	F	I	p.D448H (exon 9)	p.L483P (exon 10)	59	M	III	p.D448H (exon 9)	p.L483P (exon 10)
29	F	I	p.R535H (exon 11)	p.L483P (exon 10)	60	M	III	p.F252I (exon 6)	p.D354E (exon8) + Rec1b
30	F	I	p.L483P (exon 10)	p.L483P (exon 10)	61	F	III	p.N227S (exon 6)	Rec5b
31	M	I	p.R535H (exon 11)	Rec1a	62	M	III	p.L483P (exon 10)	p.F252I (exon 6)

^a,b,c,d^Familial cases

Thirty-one patients from 30 families had neuronopathic GD, including 10 with type 2 (16%) and 21 with type 3 (34%) GD. Four common mutations identified among Korean GD patients were p.L483P (26%, 30/116 alleles), p.G85E (16%, 19/116 alleles), p.F252I (13%, 15/116 alleles), and p.R296Q (9%, 10/116 alleles).

p.L483P was the most prevalent mutation in the 30 unrelated neuronopathic GD patients, including two homozygous for p.L483P and 11 compound heterozygotes (25%, 15/60 alleles) (Fig. 1a). Sixteen different pathogenic mutations were identified in neuronopathic GD patients (Fig. 1b).

In 31 non-neuronopathic GD patients (50%) from 28 Korean families, 15 different pathogenic mutations were identified, with p.G85E being the most common (34%, 19/56 alleles) (Fig. 1a; Table 1). This mutation is classified as a pathogenic variant in Clinvar (https://www.ncbi.nlm.nih.gov/clinvar/variation/4296). The allele frequency of p.G85E was 0.000004 in the Genome Aggregation Database (https://gnomad.broadinstitute.org/).

**Fig. 2:**
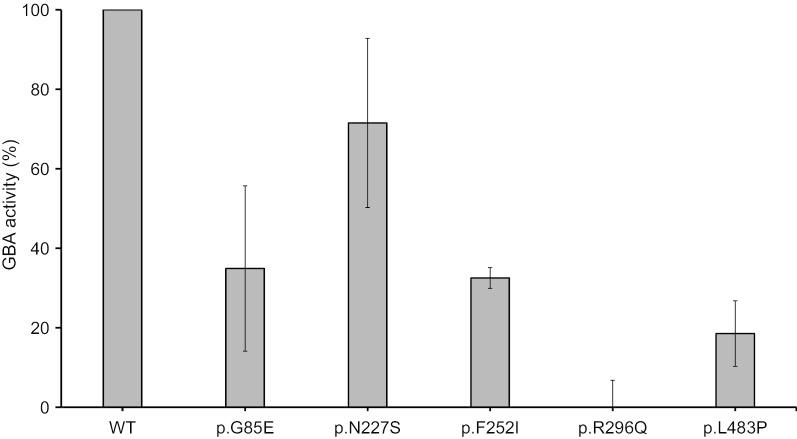
Comparison of percentages of glucocerebrosidase (GBA) activity in COS-7 cells transiently transfected with wild-type and mutant constructs

All patients harboring a p.G85E mutation were diagnosed with non-neuronopathic GD (Fig. 1a, b). Among 18 GD patients from 17 unrelated families harboring the p.G85E mutation, two unrelated patients were homozygous for p.G85E and 16 (89%) were compound heterozygotes harboring *GBA* mutations associated with neuronopathic phenotype (p.R159W, p.V230G, p.F252I, p.R296Q, p.D438N, p.L483P, and Rec8a) (Table 2). The β-glucocerebrosidase activity of mutant *GBA* p.G85E in COS-7 cells was lower (35 ± 21%) than that of wild-type *GBA* (Fig. 2).

**Table 2:** Clinical characteristic of 18 patients with Gaucher disease having homozygous or compound heterozygous mutations with p.G85E (G46E) of *GBA*

Subject no.	1^a^	2	3^a^	4	5^a^	6^a^	7	8	9^a^	10^a^	11^a^	12^a^	13^a^	14	15	16	17	18
Allele1	p.G85E	p.G85E	p.G85E	p.G85E	p.G85E	p.G85E	p.G85E	p.G85E	p.G85E	p.G85E	p.G85E	p.G85E	p.G85E	p.G85E	p.G85E	p.G85E	p.G85E	p.G85E
Allele2	p.R159W	p.R296Q	p.R296Q	Rec8a	p.D438N	p.F252I	p.F252I	p.F252I	p.F252I	p.L483P	p.L483P	p.L483P	p.L483P	p.L483P	p.I442T	p.G85E	p.G85E	p.V230G
Sex	F	F	F	F	M	M	F	M	M	F	F	M	F	M	F	M	F	M
Age at Dx	1y1m	1y 5m	1y11m	1y10m	3y	4y6m	11 y	20y	12y10m	2y3m	2y10m	2y	2y3m	57y	7y	7y	30 y	23 y
Age at ERT	1y 2m	1y 6m	1y 11m	1y 11m	3y	4y 6m	20y 3m	20y	12y11m	3y 4m	2y10m	9y	3y 4m	57y	11y7m	13y8m	31y	31y
Current age	16y1m	3y8m	14y4m	15y	11y	18y3m	40y6m	39y9m	25y7m	12y	5y1m	29y3m	19y1m	67y	31y8m	expired (19y)^b^	45y3m	49y3m
Total duration of ERT	14y11m	2y 2m	12y5m	14y1m	8y	13y9m	20y3m	19y9m	13y3m	9y	2y3m	20y3m	15y7m	10y	20y1m	5y4m	14y3m	18y3m
Hepato-splenomegaly	+	+	+	+	+	+	+	+	+	+	+	+	+	+	+	+	+	+
Skeletal involvement	+	−	−	−	+	−	+, AVN	−	+, AVN	+	+	+, OS	−	+, AVN	+	+, AVN, FX	ND	−
Bone pain at diagnosis	+	−	−	−	+	−	+	−	+	+	+	−	ND	+	+	+	ND	+
Anemia at Dx	+	+	+	+	+	+	+	−	+	+	+	+	ND	−	+	−	−	−
Thrombo-cytopenia at Dx	+	+	+	+	+	+	+	+	+	+	+	+	+	+	+	+	+	+
Height at Dx (SDS)	− 0.98	− 1.86	− 0.47	− 1.4	0.69	− 1.5	− 1.63	− 1.68	− 1.29	− 0.89	− 2.83	− 1.49	ND	− 1.88	− 2.13	− 2.24	ND	ND
Weight at Dx (SDS)	− 1.27	− 1.34	− 1.27	− 1.58	0.89	− 1.13	− 2.23	− 1.37	− 0.89	− 0.14	− 1.07	− 1.23	ND	− 0.43	− 2.42	− 1.64	ND	ND
Current height (SDS)	0.04	− 1.1	1.95	− 0.54	1.94	− 0.49	− 1.37	− 1.37	− 0.83	− 0.12	0.14	0.44	ND	− 1.88	− 0.9	0.12	ND	ND
Current weight (SDS)	0.57	− 0.24	1.01	0.4	1.86	1.02	− 0.01	0.77	0.13	0.81	− 0.17	0.3	ND	0.28	− 1.7	− 0.19	ND	ND
Operation	–	–	–	–	–	–	+, SP, CL	–	–	–	–	+, SP	–	+, HR (59y)	+, SP (7y)	+, SP (7y)	–	–

^a^The patients who included for haplotype analysis

^b^Cause of death: acute pneumococcal meningitis

*Dx* diagnosis, *ERT* enzyme replacement therapy, *SDS* standard deviation score, *AVN* avascular necrosis of femur neck, *OS* osteoporosis, *FX* fracture of bone, *SP* splenectomy, *CL* cholecystectomy, *HR* hip replacement surgery

### Three-dimensional (3D) structure of GBA and location of mutations

We determined the location of each mutation in the 3D structure of GBA (Additional file 1: Figure S1). All mutations were widely located in domains I, II, and III. The p.G85E, p.R87W, p.L483P, and R535H mutations were located in the immunoglobulin (Ig)-like domain (domain II), but they were distant from the active site, except for p.L483P, which was located in the hydrophobic core of the Ig-like domain, which may lead to disruption of the hydrophobic core and alteration of the domain folding. p.G85E, p.R87W, and R535H were identified only in type 1 GD patients. p.V54L, p.N425K, p.D438N, p.I442T, and p.D448H were located in domain I containing two disulphide bridges. p.V54, D438N, and p.I442T were identified in type 1 GD. The other 12 missense mutations were located in the catalytic domain, which is the triosephosphate isomerase (TIM)-barrel (domain III). Among mutations located in domain III, p.V211fs and p.W420* were identified in patients with the neuronopathic type. The splicing mutation (IVS2 + 1 G > A), which leads to disruption of domains I and II, was also found in type 2 GD patients.

### Clinical characteristics of patients harboring the p.G85E mutation

Eighteen patients with type 1 GD (8 males and 10 females) harbored the p.G85E mutation in at least one allele. The median age at diagnosis was 3.8 (range 1.3–57) years. Fourteen (78%) were diagnosed before 18 years of age (Table 2). Their height and weight standard deviation scores (SDS) at diagnosis were − 1.4 ± 0.8 and − 1.1 ± 0.8, respectively. Two patients (Subjects 16 and 17) homozygous for p.G85E were diagnosed at 7 and 31 years of age, respectively. Other compound heterozygote patients harboring the p.G85E mutation were diagnosed at 9.6 ± 14.4 (median 2.9, range 1.3–57) years.

Anemia and thrombocytopenia were assessed upon presentation. Initial hemoglobin and platelet levels were 10.7 ± 2.1 g/dL and 116 ± 125.9 × 10^3^/µL, respectively; however, 2 patients harboring homozygous p.G85E mutations were not anemic (Table 2).

Four patients (23%) underwent splenectomy during childhood, prior to the introduction of enzyme replacement therapy (ERT). Of the 4 splenectomized patients (Subjects 7, 12, 15, and 16), two (Subjects 7 and 16) manifested avascular necrosis (AVN) of the femur neck, while Subject 12 had osteoporosis, with a lumbar spine Z-score of − 5.4. Subject 15 had compression fractures in the thoracic spine at age 13 years. Four patients (Subjects 7, 9, 14, and 16) manifested AVN of the femur neck at ages 11, 12, 12, and 57 years, respectively. A 2-year-old female patient (Subject 11) suffered from severe bone pain and massive splenomegaly, with abnormally low T1 signal intensity in the long bone and femur neck on magnetic resonance imaging.

Following the introduction of ERT in Korea in 1994, all patients have been treated with it. Patient age at the last evaluation ranged from 3.7 to 67 years (mean 25.7 ± 17.0, median 19.0). No patients developed neurological symptoms, including abnormal eye movements, ataxia, seizures, and cognitive impairment during the follow-up period, which ranged from 2.3 to 21 years. After ERT treatment for 2.2–20.3 years (median 13.9), hematological findings improved among all patients, with mean hemoglobin and platelet levels of 13.6 ± 1.57 g/dL and 240 ± 81.2 × 10^3^/µL, respectively. Skeletal complications, including bone pain, AVN, and low bone density, had ameliorated. Symptoms of 10 patients who had complained of bone pain at diagnosis were either decreased or obliterated. Only one patient (Subject 14) with AVN finally underwent total hip replacement surgery at the age of 59 years. The bone mineral density Z-score of Subject 12 improved from − 5.4 to − 2.4. Height SDS and weight SDS significantly improved from − 1.4 ± 0.8 to − 0.3 ± 1.3 and from − 1.1 ± 0.8 to 0.1 ± 0.8, respectively (*p* = 0.001). Among patients receiving splenectomy prior to ERT, Subject 7 underwent cholecystectomy owing to acute cholecystitis and Subject 16 died of acute bacterial meningitis at age 19 years (Table 2).

### Founder effect of the p.G85E mutation among Korean patients

**Fig. 3:**
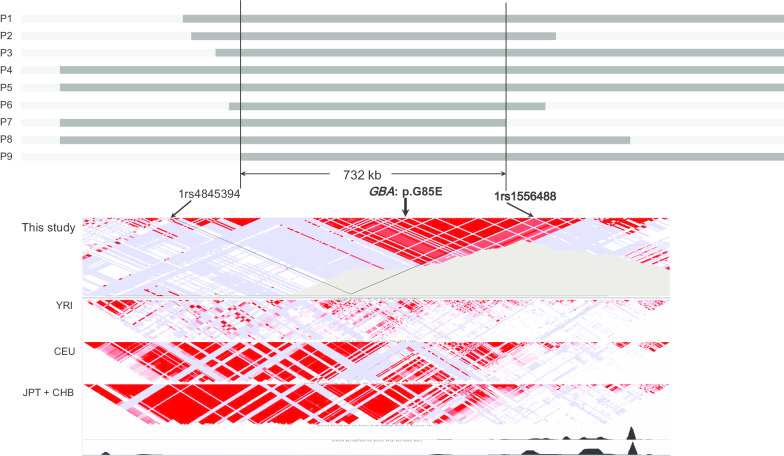
Haplotypes of the alleles with p.G85E mutation in the *GBA* gene. The gray vertical line illustrates the location of the mutation, and the horizontal black bars indicate regions of the shared haplotype in each allele. The minimum size of the shared haplotype is approximately 732 kb

On haplotype analysis, 9 patients were compound heterozygotes for the p.G85E allele with other mutant *GBA* alleles. The size of the shared haplotype was approximately 732 kbp (chromosome 1: 154,808,287–155,540,660) (Fig. 3) (Additional file 2: Table S1). All 9 alleles containing the p.G85E mutation shared a common haplotype surrounding it, suggesting a founder effect. The estimated age of the mutation was 123 generations (95% credible set; 69–248 generations); i.e., 3075 years (95% credible set: 1725–6200 years), based on an intergeneration time of 25 years [17].

## Discussion

This study assessed the mutational spectrum and phenotypic characteristics of Korean GD patients, many of whom share a unique founder mutation p.G85E. In 1996, this mutation was first reported in two Korean patients: 2- and 18-year-old boys, who presented with massive hepatosplenomegaly and isolated splenomegaly, respectively [11]. Both were non-neuronopathic and their genotypes were compound heterozygous for p.L483P and homozygous for p.G85E, respectively [11]. GBA expression of the p.G85E mutant was as low as the activity of the p.F252I mutant in neuronopathic forms [4]. The 3D structure showed that p.G85E was located in the Ig-like domain II (Additional file 1: Figure S1) and distant from the active site residues E274‒E379 [18]. In our study, patients with p.G85E presented with massive visceromegaly and bone involvement during early childhood. However, none had shown any neurologic symptoms during a relatively long follow-up period.

Genotype–phenotype correlations have been previously reported, although they have not always been consistent. Patients with at least one p.N409S allele are non-neuronopathic in Caucasians [19–23]. Furthermore, homozygous p.K118N, p.N227S, and p.G416S mutations are also non-neuronopathic. However, compound heterozygosity with null mutant alleles results in the chronic neuronopathic form (type 3) [11, 24–27].

Half of the chronic non-neuronopathic type GD (type 3) patients displayed symptoms before 2 years of age, while others exhibited initial symptoms during late adolescence [22, 28–30]. In the present study, GD patients carrying the p.G85E mutation have yet to present with any neurologic symptoms at the latest evaluation. More than half of the patients (11/18, 61%) were adults. Furthermore, Subject 4, displaying compound heterozygosity for p.G85E and Rec8a, displayed no neurological symptoms until the latest follow-up at 15 years of age. However, many GD patients with the p.G85E mutation presented with more severe hematological, visceral, and skeletal manifestations, than did typical GD patients.

These findings suggest that the p.G85E mutation may exert a neuroprotective effect, as does the p.N409S mutation in Caucasian GD patients. The p.G85E mutation was previously identified in two Chinese patients as compound heterozygotes, with one having p.N227S and the other having p.L483P, respectively [10]. The patients harboring the p.G85E and p.L483P mutations were diagnosed with type 1, whereas the other patient with p.G85E and p.N227S was diagnosed with type 3 GD; this latter patient presented with seizures and abnormal electroencephalography findings, but not oculomotor abnormalities. Recently, two Indian GD patients with p.G85E mutation were reported, but without phenotypic information [16].

In addition to having unique clinical characteristics, the p.G85E mutation is frequently observed among Korean GD patients, accounting for 16% of mutant alleles, second only to the p.L483P mutation (26%). We assessed the haplotypes of alleles with p.G85E and identified a haplotype 732 kbp in length, suggesting that a founder mutation occurred 3,075 years ago. To date, we have not encountered any case of Parkinson disease or Alzheimer disease in a *GBA* p.G85E carrier, and there have been no reports about the association between *GBA* p.G85E carriers and Parkinson disease [31]. However, we carefully counseled the patients, together with their parents, considering the late onset of Parkinson disease or Alzheimer disease [32]. Thus, we hypothesize that the p.G85E mutation may have passed through many generations within the Korean population, with positive selection for the neuroprotective effects conferred to carriers.

There are several studies on Korean founder mutations in other monogenic disorders, including p.Q258* of *STAR* in congenital lipoid adrenal hyperplasia [MIM #201710] [33], p.P240L of *CDH23* in non-syndromic hearing loss [MIM #601386] [34], and two intronic mutations of *UNC13D* (c.118–308C > T,c.754–1G > C) in type 3 familial hemophagocytic lymphohistiocytosis [MIM #608898] [35]. Due to their adjacent geographic locations, Korean, Japanese, and Chinese populations often share common mutations contributing to many autosomal recessive disorders, including c.648G > T of *G6PC* in glycogen storage disease type I [MIM #232200] [36] and p.R778L of *ATP7B* in Wilson disease [MIM #277900] [37]. Interestingly, the p.G85E mutation is predominantly found in Korean GD patients, when considering few reports of other Asian countries, such as China and India.

## Conclusion

In conclusion, this study shows the mutation spectrum of *GBA*, as well as clinical features and a founder effect of *GBA* p.G85E in the Korean population. Our data and those from a previous study [4] strongly suggest that the p.G85E mutation may behave as a neuroprotective allele in GD patients, even though their visceral and skeletal phenotypes might be severe. Further long-term, meticulous clinical follow-up may be needed to understand the neuroprotective effect of p.G85E allele and to elucidate the mechanisms underlying this effect.

## Methods

### Patients

The study cohort included 62 patients with GD from 58 unrelated Korean families diagnosed through molecular analysis of *GBA* in Korea from 2000 to 2019. Among them, 18 patients from 17 families harbored the p.G85E mutation (Table 2). Their clinical characteristics, including height, weight, visceromegaly, skeletal findings, hematological parameters, bone mineral density assessed through dual-energy X-ray absorptiometry, and neurologic symptoms, were retrospectively reviewed. This study was approved by the Institutional Research Board at each institute. Informed consent was obtained from all patients and their parents.

### Molecular analysis of the GBA gene

Genomic DNA was extracted from peripheral blood leukocytes using the Puregene DNA isolation kit (Qiagen, Hilden, Germany). Polymerase chain reaction (PCR) amplification of all coding exons and exon–intron boundaries was performed using allele-specific primers. Direct bidirectional sequencing of amplification products was performed using an ABI3130*xl* Genetic Analyzer (Applied Biosystems, Foster City, CA, USA). The long-range PCR approach was used for identification of *GBA* recombinant mutations between *GBA* and the non-functional *GBAP* pseudogene located 16 kb downstream of the functional *GBA* according to a previous report [38]. This report uses *GBA* nomenclature based on the reference sequence NP_000148.2. Traditional *GBA* nomenclature, which omits the first 39 amino-acid residues of the leader sequence of NP_000148.2, is also provided within parentheses without the p. prefix in Fig. 1.

### 3D structure modeling of GBA

We generated the 3D structure of GBA using the Cn3D Software 4.3 in National Center for Biotechnology Information (NCBI, https://www.ncbi.nlm.nih.gov/Structure/CN3D/cn3d.shtml) and the mature protein data were obtained from protein data bank with accession code 2NT1-A (https://www.ncbi.nlm.nih.gov/protein/122921009) (Additional file 1: Figure S1).

### In vitro expression of GBA mutant proteins

The β-glucocerebrosidase activity of the p.G85E, p.N227S, p.F252I, p.R296Q, and p.L483P mutant proteins was measured in COS-7 cells transfected with the pCMV6 vector. For site-directed mutagenesis, human *GBA* (NM_000157.4) cDNA (SC120080 OriGene, Rockville, MD, USA) was used as a template for the generation of mutants. *GBA* mutants, with a wild-type control, were transiently transfected into COS-7 cells using Effectene transfection reagent #301427 (Qiagen, Hilden, Germany). After transient transfection, all cells were further incubated at 37 °C in 5% CO_2_. Assays for β-glucocerebrosidase activity were performed using a standard fluorometric method. Fluorescence was detected using a fluorescence spectrophotometer (Molecular Devices, San Jose, CA, USA). Every experiment was performed in triplicate for each mutant.

### Haplotype analysis

Analysis was performed for 9 unrelated probands and their 16 family members from whom DNA samples were available and who consented to participate in the study. Genotyping of short tandem repeat (STR) and single-nucleotide polymorphism (SNP) markers was performed within the 3.8-Mb genomic region encompassing at least three recombination hotspots on both sides of the mutation [chromosome 1:153,067,786–156,925,007 (hg19)]. Eleven STR markers were selected from the UniSTS database (https://www.ncbi.nlm.nih.gov/probe/?term=Unists, Additional file 3: Table S2). PCR amplification was performed under standard conditions in a total volume of 50 μL containing 50 ng of genomic DNA. Forward primers were labeled with fluorescein amidite. The fluorescently labeled PCR products were sized by comparison to size markers following capillary electrophoresis in an ABI3130*xl* Genetic Analyzer (Applied Biosystems, Foster City, CA, USA). Data were analyzed using GeneMarker software, version 2.4.1 (SoftGenetics, State College, PA, USA). SNP marker analysis was performed using an Illumina Infinium^®^ Human Omni2.5-8 v1.3 BeadChip (Illumina, San Diego, CA, USA) including 2,372,784 SNP markers. Of these, 873 SNP markers within the designated genomic region were selected for analysis.

### Estimation of mutation age

The DMLE2.3 program (https://www.dmle.org/) was used to estimate the age of the *GBA* p.G85E mutation. The population growth rate was set as 0.025, with an intergenerational interval of 25 years [17].

### Statistical analysis

Differences between GBA wild-type and mutant enzyme activity were analyzed by the Mann–Whitney *U* test using SPSS for Windows, version 21.0 (SPSS, Inc., Chicago, IL, USA). Comparisons of height SDS and weight SDS between initial presentation and the latest evaluation were carried out using the Wilcoxon signed-rank test. Values of *p *< 0.05 were considered statistically significant.

## Supplementary information

**Additional file 1: Figure S1**. Three-dimensional structure of GBA and location of GBAmutations.

**Additional file 2: Table S1**. The list of 11 short tandem repeat (STR) markers.

**Additional file 3: Table S2**. Haplotype analysis of individuals with mutations in the GBAgene.

## Data Availability

All data generated or analyzed during this study are included in this published article and its supplementary information files.

## Abbreviations

AVN: Avascular necrosis
ERT: Enzyme replacement therapy
GBA: Glucocerebrosidase
GD: Gaucher disease
PCR: Polymerase chain reaction
SDS: Standard deviation score
STR: Short tandem repeat
SNP: Single-nucleotide polymorphism
