# Supplementary material for: The GBA p.G85E mutation in Korean patients with non-neuronopathic Gaucher disease: founder and neuroprotective effects
Source: Orphanet J Rare Dis. 2020 Nov 11;15:318. doi: 10.1186/s13023-020-01597-0 (PMC7656680; doi:10.1186/s13023-020-01597-0)
Supplement: Supplementary file 1 — Additional file 1: Figure S1. Three-dimensional structure of GBA and location of GBAmutations. [file 13023_2020_1597_MOESM1_ESM.pptx]

## Slide 1
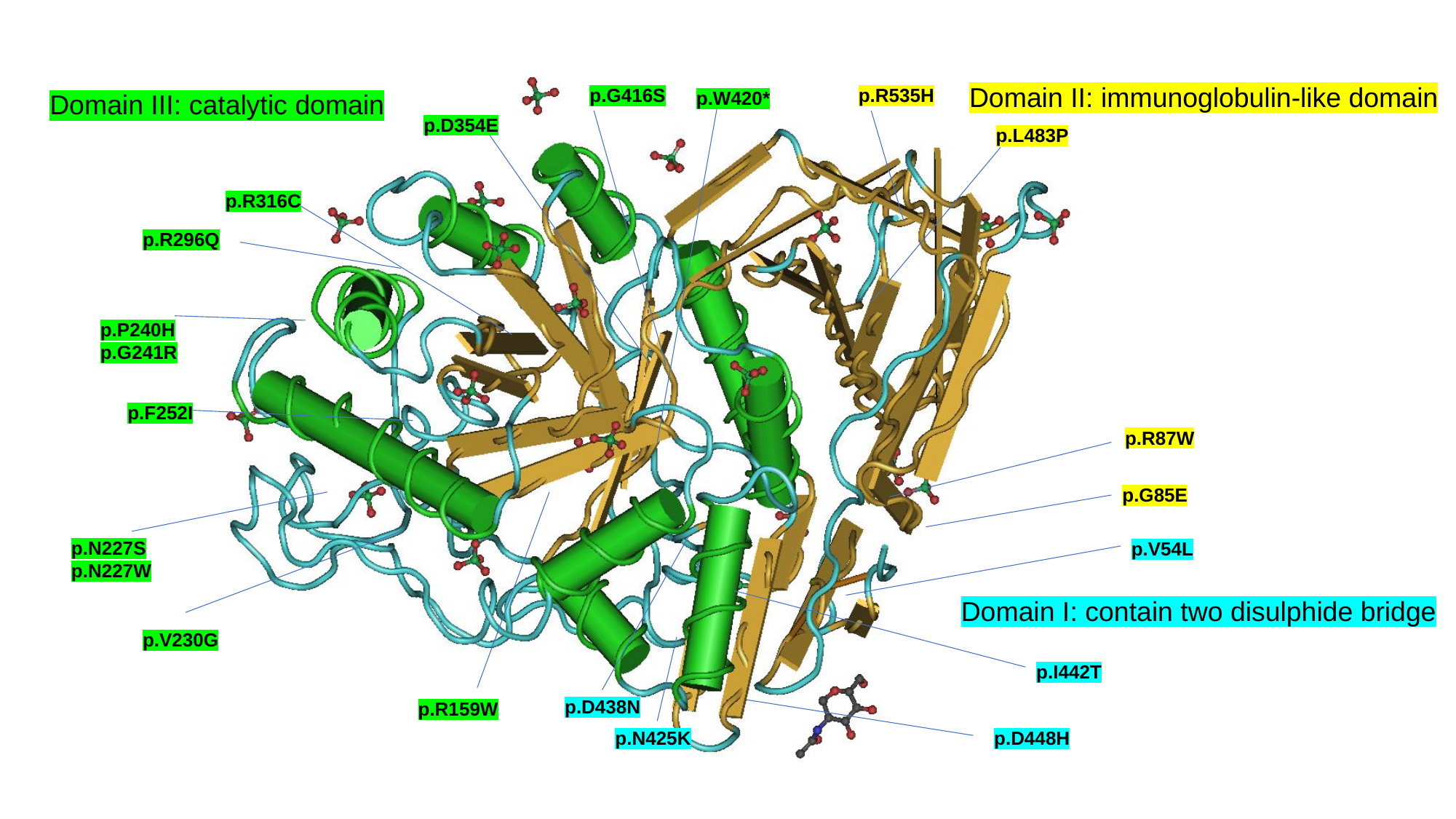

Domain II: immunoglobulin-like domain
p.R535H
p.G416S
p.W420*
Domain III: catalytic domain
p.D354E
p.L483P
p.R316C
p.R296Q
p.P240H
p.G241R
p.F252I
p.R87W
p.G85E
p.N227S
p.N227W
p.V54L
Domain I: contain two disulphide bridge
p.V230G
p.I442T
p.D438N
p.R159W
p.N425K
p.D448H
