# Supplementary material for: The GBA p.G85E mutation in Korean patients with non-neuronopathic Gaucher disease: founder and neuroprotective effects
Source: Orphanet J Rare Dis. 2020 Nov 11;15:318. doi: 10.1186/s13023-020-01597-0 (PMC7656680; doi:10.1186/s13023-020-01597-0)
Supplement: Supplementary file 2 — Additional file 2: Table S1. The list of 11 short tandem repeat (STR) markers. [file 13023_2020_1597_MOESM2_ESM.docx]

Supplementary Table 1. The list of 11 short tandem repeat (STR) markers

| STR marker  (deCODE) | Position (Human 2004) (NCBI35/hg17**)** | Position (Human 2009) (NCBI37/hg19) | Forward primer | Reverse primer |
| --- | --- | --- | --- | --- |
| D1S2344 | 1:143,020,962-143,021,254 | 146,207,887-146,208,176 | TCATGGGACTCTCCATCA | AAATACTCAGGAAATGGCCTA |
| D1S442 | 1:143,119,542-143,119,914 | 146,306,464-146,306,836 | GGTACTTAGCCTCGAAATGAGA | GTGTCACACAACTGGTTGGT |
| D1S2612 | 1:144,900,678-144,900,953 | 148,087,600-148,087,875 | GCTGTTCTTAGGGCTTTTCC | AACTTGGGCTTCTCTGCTTC |
| D1S3466 | 1:146,997,167-146,997,497 | 150,184,089-150,184,419 | ATGTCTTTGATCCTATGGAAGG | TGGGTAACAGACCCTGTCTC |
| D1S498 | 1:148,114,569-14,8114,929 | 150,184,089-150,184,419 | TTGCTGAAGGGACATAGTG | TGCTGGGTTATATCCAATATC |
| D1S2345 | 1:148,346,340-148,346,689 | 151,533,262-151,533,611 | CAAGCTCCGTCTCAAAC | CATCTTCCCAATCTACAGG |
| D1S2343 | 1:148,346,948-148,347,329 | 151,533,870-151,534,251 | GGGTGGATCACTTAAGCCT | CTAGCATATTCGTCCTGAACTAA |
| D1S2715 | 1:150,397,376-150,397,672 | 153,584,298-153,584,594 | CACAGGATTCTGCGTCTAACT | TGCTCCAAGAACTGAAGTGA |
| D1S2858 | 1:150,857,460-150,857,682 | 154,044,382-154,044,604 | AGCAGCACACTTTGAAATTG | GGCTGAGTAGTATTCCATCGC |
| D1S305 | 1:151,094,976-151,095,355 | 154,281,898-154,282,277 | CCAGNCTCGGTATGTTTTTACTA | CTGAAACCTCTGTCCAAGCC |
| *GBA* | 1:152,017,317-152,024,064 | 155,209,680-155,209,780 |  |  |
| D1S2777 | 1:152,273,433-152,273,786 | 155,460,355-155,460,708 | GCACCACGGAACTCCAGTAT | CACCACTGTGCCCAGCTAAT |

* <https://genome.ucsc.edu/cgi-bin/hgPcr>, https://www.ncbi.nlm.nih.gov/probe/?term=Unists
