# Supplementary material for: The GBA p.G85E mutation in Korean patients with non-neuronopathic Gaucher disease: founder and neuroprotective effects
Source: Orphanet J Rare Dis. 2020 Nov 11;15:318. doi: 10.1186/s13023-020-01597-0 (PMC7656680; doi:10.1186/s13023-020-01597-0)
Supplement: Supplementary file 3 — Additional file 3: Table S2. Haplotype analysis of individuals with mutations in the GBAgene. [file 13023_2020_1597_MOESM3_ESM.docx]

Supplementary Table 2. Haplotype analysis of individuals with mutations in the *GBA* gene.

| Physical position on chromosome 1 (hg37) | | Patient 1 | Patient 2 | Patient 3 | Patient 4 | Patient 5 | Patient 6 | Patient 7 | Patient 8 | Patient 9 |
| --- | --- | --- | --- | --- | --- | --- | --- | --- | --- | --- |
| rs4845394 | 154808287 | G G | G G | G G | G A | G A | G A | G A | G G | G G |
| rs121288 | 154811435 | T T | T T | T T | T T | T T | T T | T T | C T | T T |
| rs753172 | 154840516 | A A | A A | A A | A A | A A | A A | C A | A A | C A |
| rs497107 | 155130391 | G A | G A | G A | G A | G A | G A | G A | G A | G A |
| rs407203 | 155162067 | G A | G A | G A | G A | G A | G A | G A | G A | G A |
| rs4043 | 155201190 | G A | G A | G A | G A | G A | G A | G A | G A | G A |
| rs104525 | 155201235 | C T | C T | C T | C T | C T | C T | C T | C T | C T |
| rs962866 | 155206341 | T G | T G | T G | T G | T G | T G | T G | T G | T G |
| rs207556 | 155209360 | G A | G A | G A | G A | G A | G A | G A | G A | G A |
| rs77829017 (p.G85E) | 155209730 | T C | T C | T C | T C | T C | T C | T C | T C | T C |
| rs734074 | 155218446 | T C | T C | T C | T C | T C | T C | T C | T C | T C |
| rs107869 | 155235493 | A G | A G | A G | A G | A G | A G | A G | A G | A G |
| rs669688 | 155508882 | A G | A G | A G | A G | A G | A G | A G | A G | A G |
| rs1556488 | 155540660 | A C | A C | A C | A C | A C | A C | A C | A C | A C |
